# Flood survivors’ perspectives on vulnerability reduction to floods in Mbire district, Zimbabwe

**DOI:** 10.4102/jamba.v12i1.663

**Published:** 2020-03-09

**Authors:** Blessing Mucherera, Emmanuel Mavhura

**Affiliations:** 1 School of GeoSciences, University of Edinburgh, Edinburgh, United Kingdom; 2 Geography Department, Bindura University of Science Education, Bindura, Zimbabwe

**Keywords:** disaster, floods, flood-based farming, hazard, Mbire, vulnerability

## Abstract

Disasters result from the interactions of hazards and vulnerability conditions. Considering the perspectives of survivors of a disaster event is critical for reducing the progression of vulnerability conditions. The Mbire community in Zimbabwe is facing increasing threats from recurring high- and low-magnitude floods that manifest themselves in the disruption of livelihoods and destruction of crops and infrastructure. This study, therefore, explored the perspectives of flood survivors on vulnerability to floods and examined their vulnerability-reduction measures. Using an interpretivist approach to knowledge generation, a sample of 51 research participants provided data through interviews, a focus group discussion and field observations. Results showed that shortage of land, flood-based farming practices, poverty and climate change, amongst others, are the key drivers of the smallholder farmers’ vulnerability to floods. The most affected groups of people include women, children and the elderly. To reduce their vulnerability, the smallholder farmers mainly rely on traditional flood-proofed structures built on stilts, dual home system and indigenous flood forecasting. The study proposes six policy implications to reduce vulnerability to floods. These include diversifying rural livelihoods beyond the farming sector, investment in irrigation infrastructure, increasing access to financial resources, constructing human settlements away from floodplains, enforcing environmental laws regarding flood-based farming and community education on the long-term negative impacts of recession farming. The implementation of these policy recommendations can contribute to community resilience to flood disasters.

## Introduction

The term ‘vulnerability’ is widely used in physical as well as social sciences. As a result, a dozen scholarly definitions of vulnerability have emerged across disciplines and practices (Kelman et al. 2016; Mavhura 2018). To promote a common understanding of vulnerability, the United Nations Office for Disaster Risk Reduction (UNISDR 2009) defined vulnerability as the characteristics and circumstances of a community, system or asset that make it susceptible to the damaging effects of a hazard (United Nations 2016). This perspective views vulnerability as the degree to which a system reacts adversely to actual or perceived threat(s) (Gain et al. 2015). Vulnerability is usually determined by a combination of physical forces and socio-economic processes of the human–environment system (Kusenbach, Simms & Tobin 2010). The fundamental question underlying vulnerability analysis is: ‘How do natural hazards affect a society?’ To answer this question, Armaș and Gavriș (2013) viewed vulnerability as a significant determinant of disasters than hazards themselves. They distinguished between social and physical vulnerability. Their view was supported by Gain et al. (2015) and Kusenbach et al. (2010) who considered social vulnerability as the susceptibility of humans and the conditions necessary for their survival and adaptation, whilst physical vulnerability is the extent to which a system is exposed to adverse effects of a hazard and is (un)able to adapt to its impacts. Social vulnerability is widely viewed as a product of social inequalities that increases the susceptibility of population groups to harm and reduce their ability to respond to disturbances (Armaș & Gavriș 2013; Siagian et al. 2014). Individual characteristics of people, including their age, health, income, type of dwelling units and employment, describe the social construction of vulnerability. The degree to which communities are vulnerable to hazards is not solely dependent upon their exposure to a hazard but also on their demographic and socio-economic characteristics (Berkes 2007; Solangaarachchi, Griffin & Doherty 2012). Such factors are independent of the hazards triggering their vulnerability but greatly influence their capacity to prepare for, respond to and recover from hazards or disasters. Because many societies live under different social, economic, political, cultural and institutional settings, they have varying levels of vulnerability.

In rural southern Africa, vulnerability to hydro-meteorological threats is greatly influenced by place-based environmental, socio-economic, political and climatic conditions (Shiferaw et al. 2014). Amongst all observed natural and anthropogenic adversities, floods and drought are the most recurrent hazards in Africa (Masunungure & Shackleton 2018). Floods increase the vulnerability of rural households that mainly depend on rain-fed agriculture and livestock production (Mavhura 2018) but with limited infrastructure and institutional support (Muzamhindo et al. 2015). The vulnerability of smallholder farmers in southern Africa also comes at the backdrop of projections of increased flood frequency and intensity (Klein et al. 2014). Their exposure is worsened by high levels of sensitivity of social-ecological systems and the limited capacity of institutional actors who respond to emerging threats (Jiri & Mafongoya 2018). Coupled with high poverty and limited employment opportunities, these conditions amplify smallholder farmers’ vulnerability to hydro-meteorological hazards (Mavhura 2019). Given these conditions, vulnerability studies enable exploration of societal capacities and exposure in space and time. The combination of exposure and capacity allows the concept of vulnerability to link across problem areas and geographical levels. Whilst changes in the environment are a source of exposure, sensitivity to these is the basis for defining the degree to which specific places are more or less vulnerable than others (Muzamhindo et al. 2015). Therefore, this study sought to explore the perspectives of flood survivors on vulnerability to floods in Mbire district, Zimbabwe. It also examined the flood survivors’ vulnerability reduction measures and recommended policy implications to reduce vulnerability to floods. The rest of the article is organised into seven sections. After this Introduction, the next section reviews frameworks for vulnerability analysis and the factors influencing susceptibility to floods, and the vulnerability reduction measures. Then a description of materials and methods used to gather data follows. The results and their discussion mark the section before the conclusion and policy implications of the study.

## Literature review

### Frameworks for vulnerability analysis

**FIGURE 1:**
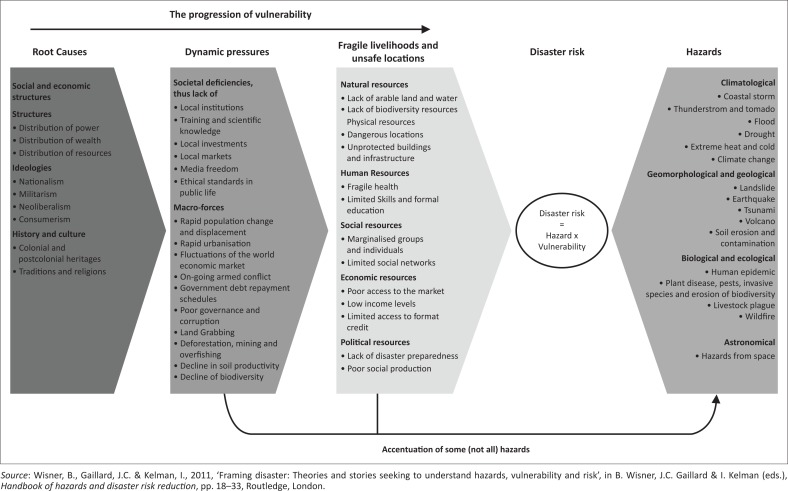
The pressure and release model.

Several frameworks advance the analysis of vulnerability to hazards. These include Cutter, Boruff and Shirley’s (2003) hazards of place model and Turner et al.’s (2003) place-based approach. However, this study is informed by the Pressure and Release (PAR) model developed by Blaikie et al. (1994). Figure 1 is an improvement of the original model developed by the same group of scholars. The PAR model views disasters as the intersection of two significant forces: the processes generating vulnerability on the one hand, and the natural hazard event on the other hand. Then, the ‘release’ idea is factored to conceptualise the reduction of disaster, that is, to relieve the pressure, vulnerability has to be reduced (Kusenbach et al. 2010). Vulnerability is understood within three levels of progression, namely, root causes, dynamic pressures and unsafe conditions. The root causes are a set of well-established, wide-spread processes within a community (Wisner, Gaillard & Kelman 2011). The major underlying causes that yield to multiplication of vulnerability over time are economic, demographic and political processes. These affect the allocation and distribution of resources between different groups of people. The root causes, such as limited access to power and resources, as well as ideologies, build dynamic pressures on communities. The pressures are processes and activities that channel the effects of root causes both temporally and spatially into unsafe conditions. They are societal deficiencies that include lack of institutions, markets and scientific knowledge as well as macro-forces such as land-grabbing, deforestation and decline in soil productivity. Unsafe conditions are the specific expression of vulnerability of a population in time and space in conjunction with a hazard. These include living in flood-prone locations and engaging in fragile livelihoods. Fragile livelihoods encompass all resources that fail to sustain people’s basic needs such as food, shelter, clothing, cultural values and social relationships. The progression of vulnerability builds up pressures on communities that can be released by vulnerability reduction measures all along the causal chain (Wisner et al. 2004).

The PAR model makes a significant contribution to the conceptualisation of flood disasters in this study. The flood disasters occur when processes generating vulnerability conditions intersect with community exposure to flood hazards. The model also identifies the drivers of vulnerability to floods and informs areas in need of policy interventions. It further helps to explain why some communities live in unsafe areas; why different groups experience different impacts from floods; and why people have different capacities to cope with or adapt to flood events (Blaikie et al. 1994). Based on this model, if Mbire district invests in flood vulnerability reduction, it may reverse the ‘progression to vulnerability’ into the ‘progression to safety’ that builds a resilient community.

However, the PAR approach has its shortfalls when analysing vulnerability to hazards. The fundamental weakness is that different elements of this framework are dynamic in that they are subject to constant change. In dynamic environments, firstly, it is hard to differentiate between the causal links of different dynamic pressures on fragile livelihoods and unsafe locations, and the impact of root causes on dynamic pressures (Wisner et al. 2011). Secondly, the PAR model puts a heavy emphasis on national and global pressures, although many dynamic pressures and unsafe conditions might also be determined by local conditions.

### Factors that increase smallholder farmers’ exposure and susceptibility to floods

Reviewed literature shows some generic social indicators represented by various variables that can increase vulnerability to floods in local populations. Age, gender, poverty and settlement in floodplains are likely to determine the vulnerability of communities and individuals to floods.

#### Age of flood-affected persons

Age is commonly regarded as a prominent vulnerability indicator to flood hazards (Raphael & Meldrum 1994; Paton 1996 cited in Miller, Paton & Johnston 1999), with children and the elderly, in general, being considered to be more at risk (The Sphere Project 2011). Children aged 15 years and below and persons aged above 65 years tend to be more vulnerable to flood hazards (Lee & Vink 2015). The vulnerability of these two age groups increases through drowning and their inability to swim because of feeble bodies in the old and lack of skills in infants (Rufat et al. 2015). Scholars recommend the use of risk awareness, disaster education and evacuation of people at risk as measures to reduce flood vulnerability amongst the children, the elderly, their families and the community in general (Lee & Vink 2015).

#### Gender of flood-affected persons

Women are more vulnerable to flood hazards because of cultural practices that reinforce significant social inequalities between women and men (Ciampi et al. 2011). Nabegu (2014) has observed that more females (72%) than males (28%) died from flooding in Kano state, Nigeria, because of gendered roles. Vulnerability to flood hazards also increases when women take care of children and the sick, prepare food and do all farming activities, whilst men do the formal work (Islam et al. 2017). In such situations, cultural norms such as wearing long dresses that make it difficult for women to swim vigorously limit their ability to take early action (Hunter et al. 2016). In order to reduce vulnerability to floods, some scholars recommend increasing access of women to control of flood information, financial and technical resources as well as stronger participation in community decision-making processes (Ciampi et al. 2011). Also, equipping women with swimming skills can save them during flood events (Hunter et al.2016).

#### Poverty levels of flood survivors

Although poverty is multidimensional, many scholars emphasise on the monetary dimension, which uses income or consumption expenditure as measures of household welfare (Lekobane & Seleka 2017). These two are justified measures of welfare because they indicate an individual’s ability to obtain goods and services. Income is vital to build decent shelter and modern flood-proofing structures. Lack of income to purchase materials for constructing proper dwellings or retrofitting existing structures increases household vulnerability to floods (Baiyegunhi & Fraser 2010; Kikwasi & Mbuya 2019). Poor people are also vulnerable to floods because of their difficulties in accessing critical resources and lifelines, such as communication and transportation (Fothergill & Peek 2004). Owing to limited income for flood mitigation, preparedness, relief and recovery efforts (Long 2007), poor people are more likely to live in unsafe and substandard housing. The practice of settling in unsafe places exposes the occupants, their livelihoods and property to floods. In general, poverty weakens poor people’s ability to respond effectively to disasters. Meaningful inclusion of the poor in decision-making may reduce their flood vulnerability (Adger 2006). Social protection measures, such as insurance, can reduce the vulnerability of the poor by transforming and protecting their livelihoods (Moser & Gonzalez 2016).

Whilst flood vulnerability can be reduced by settling off flood zones, people continue to live and work in such unsafe places because they seek sustenance and want to exploit the gains of affordable transport, commerce and agriculture-related water proximity (Morton & Olson 2018). For instance, over 20 million people and 40 ethnic groups are settled in the Niger Delta floodplain in Nigeria because of fishing and farming opportunities (Bariweni, Tawari & Abowei 2012). Notwithstanding the economic benefits of settling in floodplains, the Niger Delta case takes the ‘naturalness’ out of disasters because people have settled in harm’s way. Settling in harm’s way increases the risk of loss of life, livelihood and property. In such areas, structural measures, such as constructing levees, dykes, walls and retrofittings, can reduce flood risk, whilst non-structural mitigation measures, including insurance, land-use planning and flood forecasting for early warning, may reduce the flood impact (Kundzewicz et al. 2018).

### Flood vulnerability reduction measures

The uptake of adequate precautionary measures to reduce flood vulnerability can save life, livelihood and property. Some possible flood vulnerability reduction measures include land-use control through legislation, flood proofing, forecasting and warning systems, and community preparedness (Hyndman & Hyndman 2011).

#### Land-use control through legislation

Land-use control through legislation serves to reduce danger to life, property and livelihood when high waters inundate the floodplains or the coastal areas. Regulating building design, siting and zoning have been used for the settlement of populations on floodplains for many decades (Tasantab 2019). In Zimbabwe, five main pieces of legislation have been enacted to control land use and thereby reduce human vulnerability to hazards. *The Environmental Management Act (Chapter 20:27) of 2002* (Government of Zimbabwe, 2002a) calls for the protection of beds and banks of public water sources, by prohibiting the construction of buildings anywhere near the water body. *The Housing Standards Control Act (Chapter 29:08) of 1972* (Government of Zimbabwe, 1997) regulates building codes and calls for the demolition of unsafe or unhealthy habitats. *The Regional Town and Country Planning Act (Chapter 29:12) of 1976* (Government of Zimbabwe, 1998) regulates densities by stipulating that buildings should conform to the plan of the local planning authorities mandated to improve sites and reduce vulnerability. The complementing *Rural District Councils Act (Chapter 29:13) of 1988* (Government of Zimbabwe, 2002b) and the *Urban Councils Act (Chapter 29:15) of 1997* (Government of Zimbabwe, 2002c) empower local authorities to remove any public water source encroachments. The thrust of these pieces of legislation is at ensuring the safety of people by reducing their vulnerability hazards. However, weak government enforcement, coupled with community reluctance to comply with the legislation, has limited the success of these policies (Mangena 2014; Naome, Rajah & Jerie 2012).

#### Flood proofing

Flood proofing describes structural measures taken to protect building facilities from flood water (World Meteorological Organization 2012). Temporary flood-proofing measures on buildings and dwellings include blocking or sealing entrances or windows and the use of sandbags or inflatable tubes to keep flood waters away (Hyndman & Hyndman 2011). These were used in New Orleans, during the 2005 Hurricane Katrina after the failure of Mississippi levees. Permanent flood-proofing measures include use of hazard-resistant designs, such as raising living or working spaces high above the possible flood level (Attems et al. 2019). This study investigated the application of these flood-proofing measures in reducing the vulnerability to floods in Mbire district.

#### Flood forecasting and warning systems

Flood-control measures can be used in combination with flood forecasting and warning systems. According to Bariweni et al. (2012), flood warning is to advice ahead of time on conditions that are likely to cause flooding to property and a potential risk to life. Thus, communication systems for public warning must be well planned so that other procedures such as evacuation could be initiated. Ways to disseminate warnings include the Internet, radios, televisions, cell phones or telephones, warning sirens or bells, public address systems, and at the village level by bicycle and on foot (Inayath 2016). It is also imperative to consider how indigenous knowledge systems have been applied. This is because, over the years, disaster-affected communities have evolved their own coping strategies and early warning systems through the use of indigenous knowledge (Chanza & De Wit 2016). For instance, in Muzarabani district, Zimbabwe, easterly winds indicate an imminent storm, which results in severe flooding as well as torrential rainfall (Ncube-Phiri, Mudavanhu & Mucherera 2015). It is, therefore, of interest to find out which systems are being applied in Mbire district and how the early warning information is being disseminated for flood vulnerability reduction.

#### Community preparedness

The International Federation of the Red Cross and Red Crescent Societies (2000) defined preparedness as:

[*M*]easures taken to prepare for and reduce the effects of disasters (t)hat is, to predict and – where possible – prevent them, mitigate their impact on vulnerable populations, and respond to and effectively cope with their consequences. (p. 6)

Thus, preparedness encompasses all pre-disaster activities undertaken within the context of disaster risk management. When households adequately prepare themselves before the disaster, lives could be saved; and injuries, property damage and the psychological pain and stress associated with hazard occurrences could be reduced (Schlör, Venghaus & Hake 2018). Examining aspects of community preparedness, including emergency plans, resource availability, evacuation, search and rescue, flood knowledge and awareness, is vital for flood-prone places (Tanwattana 2018). The advantage of active community preparedness is that it addresses the real needs of the community as opposed to perceived needs (Abarquez & Murshed 2004). Therefore, the actual problems could be addressed with appropriate local interventions.

## Materials and methods

### Study setting

**FIGURE 2:**
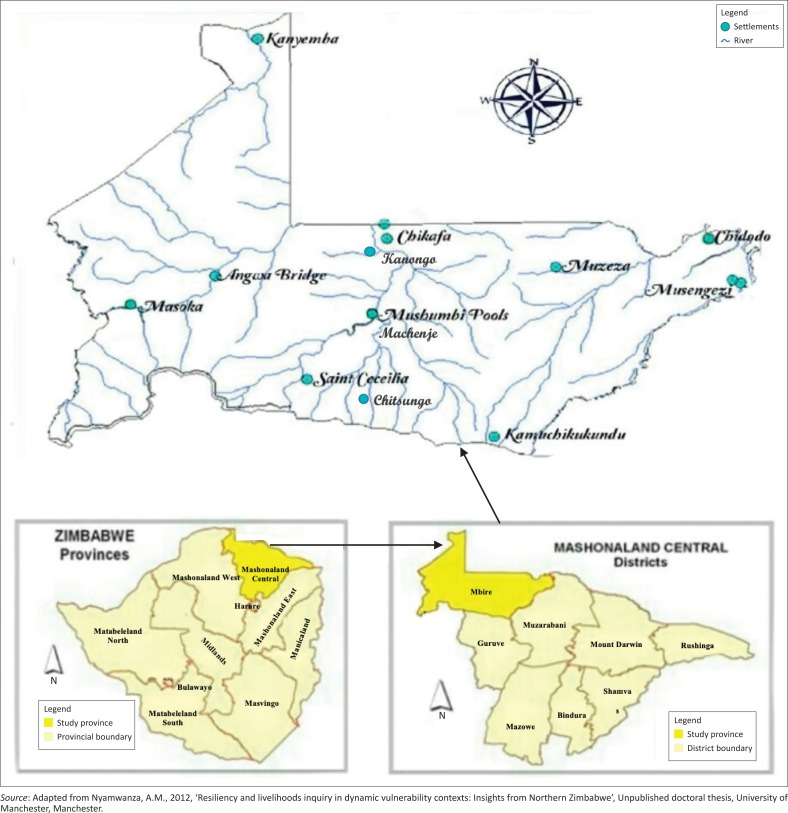
Maps depicting Mashonaland Central province, Mbire district.

This study was conducted in Mbire district, in the middle of the Zambezi Valley, in Mashonaland Central Province, Zimbabwe (Figure 2). The district lies in ecological regions IV and V, characterised by low annual rainfall, which in most parts is below 450 mm and too erratic even for drought-resistant crops (Mavhura, Manatsa & Mushore 2015). The primary economic activity in Mbire district is rain-fed smallholder farming. Major crops grown include maize, small grains, cotton and edible dry beans. Owing to prolonged dry spells and drought conditions, many smallholder farmers have settled on the floodplains where they practice flood-recession farming. The floodplains provide water for livestock and domestic uses as well as residual moisture and fertile alluvial soils for their crops. Major rivers along which flood-based farming takes place include Hunyani (also known as Manyame), Dande and Angwa. Small-scale livestock production (cattle, goats and sheep) supplements rain-fed smallholder farming (Mavhura 2019). However, both crop and livestock production is constrained by floods and droughts, rendering these two as very fragile livelihood activities. Settling on floodplains also exposes the smallholder farmers to flood risk. In this way, development and disasters closely link to each other: Failed development leads to disasters.

The history of floods in Mbire district dates back to the middle of the 1st millennium AD (Nyamwanza 2012). Since then, floods have been occurring nearly yearly, whilst their severity and impact have varied from one season to another. The 2015 floods were the most recent high-magnitude floods that encroached onto new areas that were previously not susceptible to flooding. These floods caused insurmountable damages to transport and housing infrastructure, crops and water, sanitation and hygiene (WASH) facilities. About 498 households were directly affected by the floods: 109 housing units were rendered uninhabitable, more than 60% of the WASH facilities were destroyed, about 80% of crops were washed away, livestock and farming implements were lost, and about three-quarters of the roads and bridges were destroyed (Mbire District Civil Protection Department 2015). On the one hand, the destruction of WASH facilities triggered the outbreak of water-borne diseases, mainly cholera and typhoid. On the other hand, the destruction of roads and bridges meant that the communities became inaccessible for emergency response. It is against these problems that this study sought to explore the flood survivors’ perspectives on vulnerability reduction.

### Methods

A qualitative research methodology was used to collect data on the perspectives of flood survivors on vulnerability reduction to floods. Qualitative research is ‘multi-method in its focus, involving an interpretive, naturalistic approach to its subject matter’ (Gall, Borg & Gall 1996:28). This study adopted an interpretive position on the assumption that the individuals who participate in it construct the social reality. The interpretivist approach to knowledge-generation recognises the importance of subjective human creation of meaning, and the task has been to ‘interpret the range of constructions and meanings that present themselves in participants’ perceptions and experiences’ (Crabtree & Miller 1999:10).

The research tools used included in-depth interviews, a focus group discussion (FGD) and field observations. In-depth interviews involved 40 purposively selected key informants from Mbire Rural District Council, the district administrator, ward councillors, Lower Guruve Development Association, Red Cross Society of Zimbabwe and survivors of the 2015 floods drawn from the most affected wards: Kanongo, Mushumbi, Chikafa and Chitsungo. The key informants consisted of 24 women and 16 men. One FGD involving 11 village heads from the most affected wards was conducted. Eleven people were a manageable number because a very small group could have suffered from intra-group dynamics that exert a disproportionate effect. Such dynamics could have led to non-participation by some members and dominance by others. At the same time, a large group could have become unwieldy and hard to manage, denying a voice to inarticulate members when disagreements and conflicts arise. Hence, the focus group method empowered participants, including the less educated, to speak out and to voice their opinions. Both the interviews and FGD centred on the processes generating their vulnerability to floods and the vulnerability reduction measures. Field walk-through analyses were also conducted to have an appreciation of the extent of the flood vulnerability, as propounded by the flood survivors. Key informants from the most affected villages led the field observations in places such as the Hunyani riverbanks, field crops, settlements and other infrastructure that were damaged by the 2015 floods. Engaging in overt semi-structured interviews in natural settings allowed gathering of data that illuminated and explained the community’s flood vulnerability in a less pre-determined manner. Observational data were triangulated with interview and focus group data.

### Trustworthiness

The article contains personally collected data through in-depth interviews, FGD and field observations. The collected data were verified with field participants in addition to triangulation that enhanced credibility and dependability.

### Credibility and dependability

Multiple data sources were triangulated and member-checked to ensure study credibility and dependability.

### Limitations of the study

Some key local government officials were busy or not available, and their interviews had to be rescheduled several times during the data collection period. Patience and flexibility were the virtues that helped in rescheduling of the interview dates and times.

### Ethical considerations

Firstly, permission to carry out the research was sought from the district administrator, ward councillor and village headmen. Informed consent was then sought from the respondents whilst their anonymity, confidentiality and privacy were observed. Informed consent to use personal images was also sought from research subjects. Full details of the research and its intended purpose were disclosed and explained to the respondents and they were not deceived in any way.

## Results and discussion

### Perspectives of flood survivors on vulnerability to floods

Of the 40 key informants, the majority (50%) opined that the causes of vulnerability to floods in Mbire were rooted in limited access to arable land, whilst 30% blamed their vulnerability on acts of God, ancestral spirits or nature, including climate change/variability. However, 20% of the informants identified flood-based farming practices as the root cause of their vulnerability. Key informants explained that limited access to arable land was a significant cause because the Mbire community had settled in or indulge in farming along unsafe riverbanks and floodplains of Hunyani, Dande and other rivers where they are exposed to river flooding. This finding resonates with the PAR model, especially ‘unsafe conditions’ that cause vulnerability. The community has increased exposure, leading to the destruction of crops, settlements and other household property. The destruction of their crops results in limited savings and income to build stronger houses, evacuate promptly using cars and reconstruct their homes without external assistance. Similarly, Mavhura, Manyena and Collins’ (2017) observed that poor people’s vulnerability emanates from their difficulty in accessing critical resources and lifelines.

However, those who believed that their vulnerability to floods was an act of God or nature asserted this by saying:

‘There is a mythical powerful mudzimu wetsunguni snake (a snake viewed as an ancestral spirit). After a while, the snake sends firewood to its in-laws downstream of Zambezi River. The flood waters help it to carry these logs and, in that event, it also washes away other things like houses and livestock’. (Female, villager, 53 years)

However, some of the survivors did not share this view. Instead, they viewed their vulnerability as a divine punishment for moral decadence such as prostitution, violence and drug abuse in the community. A few others explained that flooding was a natural event that coincided with human settlement or their livelihoods in floodplains. These respondents also linked their vulnerability to climate change and variability. All these perspectives seem to exonerate the villagers from their decisions to settle or farm along floodplains. As Paidakaki (2012) has argued, if disasters are acts of God or nature, then nothing can be done about them as people adopt a fatalistic approach. The fatalistic approach reduces the impetus of investing in disaster risk-reduction and limits public action in disaster preparedness. The projected increase in flood intensities and frequencies in southern Africa (Spear et al. 2015) highlight the need to strengthen disaster preparedness of the communities to reduce their vulnerabilities.

**FIGURE 3:**
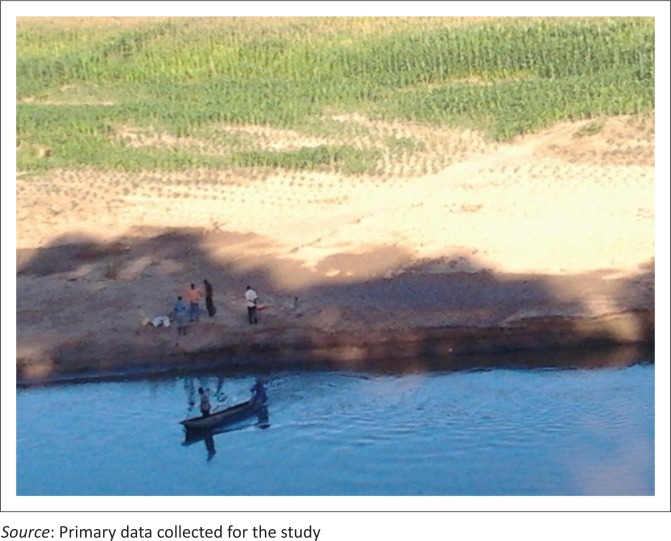
Maize crops grown along the Hunyani riverbank.

The respondents who identified flood-based farming practices narrated stories of how the farming systems were exposing the villagers to flood risks. They explained that crop cultivation along floodplains was wiping off native tree species that firmly hold the soil. The farming system was causing heavy siltation, which exposed communities to flash floods. Fields’ observations revealed that large tracts of crop fields were located along Angwa, Hunyani and Dande riverbanks and, in some instance, on the islands of major rivers. Vast amounts of forests were cleared along the riverbanks to pave way for flood-based farming. Maize is the main crop grown along the Hunyani riverbank (Figure 3), whilst other crops are grown at a small scale. Temporary shelters were also seen close to rivers or on the islands. These shelters were used for housing smallholder farmers engaged in flood-based farming and guarding their crops from wild animals. One of the key informants who was a village head justified the flood-based farming practice in this way:

‘Although floodplain farming is increasing our vulnerability to floods, it is beneficial to us in another sense. The wet fertile soils in the stream bank allow us to grow maize, a practice that is locally known as *mudzedze*. The fertile alluvium left by the floods gives us the impetus to keep on practising *mudzedze*’. (Male, village head, 66 years)

The FGD revealed that *mudzedze* farming is a type of recessional agricultural practice in which crops are planted at the end of the rainy season (around March/April), whilst harvesting occurs around September/October. The timing makes the practice an off-rainy season farming activity. Instead of irrigating their crops, the smallholder farmers make use of residual moisture left by floods during the previous rainy season. During the rainy season (December to March), the smallholder farmers also grow food crops and cotton using normal rains. As a result, the farmers in Mbire practice a dual cropping season. The FGD participants also highlighted that the harvest from flood-based farming was at times much better than that from rain-fed agriculture.

Walk-through analyses revealed many smallholder farmers had settled along floodplains where exposure to floods is high. The villagers had two homesteads each: one in the floodplain (locally known as *kugowa*) and another one outside the floodplain. When asked why they had a dual home system, one of the flood survivors explained as follows:

‘The main reason for settling in the floodplains is to protect crops from wild animals such as elephants and hippopotamuses. Farming in the floodplain is carried out all year round using residual moisture, while outside the floodplain crop failure is certain due to long dry spells and drought that characterises our region’. (Female, villager, 45 years)

Flood-based farming is also a common practice in Muzarabani (Zimbabwe), Malawi and west Africa (Mavhura 2017; Puertas et al. 2015). Although the farming system is an adaptive strategy to counter drought and long dry spells, it silts up river systems and exposes smallholder farmers to floods. Therefore, flood-based farming should be discouraged using multiple strategies. As propounded by Mavhura (2018), firstly, the existing environmental laws by the responsible authorities need enforcement, and secondly, a deliberate education of the community on the long-term impacts of the practice needs to be promoted.

When asked about the population groups that were most vulnerable to floods, all the key informants pointed at women, the elderly and children. The FGD also supported the views that women were vulnerable to floods because of cultural practices that reinforce significant social inequalities between men and women. The Shona culture in Mbire demands that women take care of children, the sick, prepare food and do all subsistence farming activities, whilst men seek formal employment. One female flood survivor said:

‘As women, our role makes us vulnerable to floods. During floods, we try to make sure that all children, older people and even the sick are safe. In that process, we suffer the brunt’. (Female, villager, 42 years)

The focus group further explained that in some remote villages of Mbire, women have to fetch water from distant places; accompany young children to distant schools; and walk long distances to access healthcare. These chores place a considerable burden on women who do not readily access flood early warning information. Furthermore, female participation in community preparedness and decision-making fora is limited. Women also have a more difficult time during recovery, often because of unemployment and low incomes. Another female narrated:

‘We take children to school and health centres while our men attend all the important meetings. At home, we do all the household chores; we even fetch water and firewood and cook for everyone. We have no time to perform paid work, attend meetings that affect our well-being and to access flood early warning information. Often we are taken by surprise when floods occur, and we are not sure what action to take’. (Female, villager, 25 years)

When asked to explain how the older people were vulnerable to floods, focus group participants narrated stories that justify the inclusion of this demographic group. Firstly, the elderly are unable to withstand the trauma associated with flood disasters. They also face difficulties in receiving flood warning when they live alone in isolated huts. Others have mobility constraints to flee from impending floods. Most of the older people in Mbire also lack financial savings to enable them to recover from floods. All these limit their capacity to deal with flood vulnerability. These conditions are consistent with vulnerability literature asserting that women, children and the elderly are amongst the most vulnerable groups in disaster-prone places (Lee 2014; Mavhura et al. 2017; Siagian et al. 2014). The findings are also consistent with the PAR model’s unsafe conditions as women, children and the elderly are special groups at risk.

**FIGURE 4:**
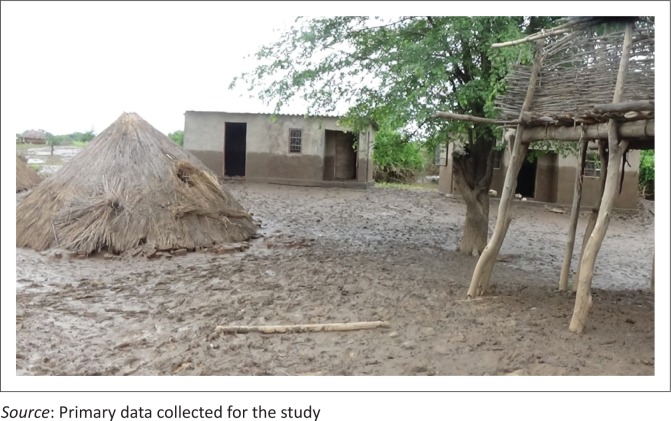
Destroyed weak mortared houses, intact cement-built houses and granaries on stilts.

Interviewees and the focus group agreed that high poverty in the district had a significant share in their increased vulnerability to floods. They explained that most smallholder farmers were unable to invest in farming and build proper structures against floods because of poverty. They narrated stories of a few sturdy houses built with cement that withstood the devastating floods (Figure 4). Other dwelling structures of wooden poles and grass succumbed to the floods. When asked how many households could afford to build proper structures using bricks and cement, focus group participants estimated that more than three-quarters of the households in the district were extremely poor, with no capacity to build proper houses.

Poverty has been observed as one of the critical drivers of vulnerability to hydro-meteorological threats in both the developed and the developing world (Armas et al. 2017; Cutter et al. 2009; Mavhura et al. 2017; Siagian et al. 2014). In the nearby district of Muzarabani, Zimbabwe, Ncube-Phiri et al. (2015) observed that high poverty rate increased community vulnerability to disasters in four related ways. Firstly, the villagers could afford only very few household assets, thus impeding effective disaster preparedness and recovery. Secondly, poverty limits livelihood diversification, which may reduce community overdependence on rain-fed smallholder farming. Thirdly, impoverished villagers are drawn into unsafe low-lying areas with the hope of eking a living. Nevertheless, by doing so, they further exposed themselves to floods; and lastly, because of high poverty, villagers cannot afford flood insurance. Therefore, poverty is closely linked with the vulnerability to flood hazards.

In Mbire, households with high disposable income are better placed to survive when faced with floods. Such households invest in sturdy dwellings and irrigation farming infrastructure. Through these flood vulnerability reduction investments, they are less exposed to hazard impacts. As depicted in the PAR model, poverty is, therefore, a root cause, which impedes upon the community’s preparedness, response and recovery efforts. Low income, and a consequent lack of diversified livelihoods, push the community into a dynamic pressure consisting of limited investment in flood vulnerability reduction measures. This dynamic pressure leads the community into an unsafe condition of residing in dangerous structures located in low-lying areas, without the protections of flood risk insurance.

### Flood survivors’ vulnerability reduction measures

Key informants reported that most flood survivors in Mbire had developed their vulnerability reduction measures. These measures include the construction of raised structures, dual home system, the use of plastic containers and growing of drought-resistant crops. However, not all the flood survivors use all these measures at one time. Instead, the adoption of these measures is a function of the level of each household’s vulnerability and ability to absorb flood shocks.

**FIGURE 5:**
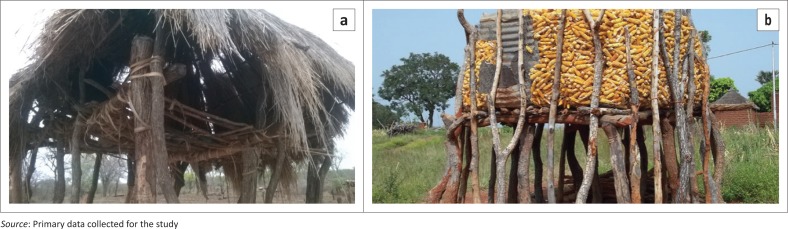
Multipurpose structures raised above the flood-predicted level. (a) Raised tower structure (b) Raised structure for storing food.

To save people’s lives, the focus group revealed that they construct raised granaries and other dwellings in floodplain fields. Most dwellings are built on stilts to prevent flooding during the rainy season. The purpose of the structures is three-fold: (1) they act as accommodation; (2) they serve as towers to look for wild animals that may destroy their crops; and (3) they are storerooms of farming implements, grain and household assets. Figure 5a shows a structure that is used as a tower, whilst Figure 5b shows another structure for storing maize. Focus group participants indicated that the building of such structures is a typical indigenous practice throughout the district. This practice is part of the community’s indigenous knowledge system for disaster risk reduction. One key informant explained the purpose of raising the structures in this way:

‘We build granaries on stilts so that when the floods occur, they do not inundate our food stocks and seed banks stored in them. The raised structures provide a relative measure of security during floods. They keep us safe from crocodiles, especially at night when we are sleeping. During the day, the same structures enable us to see afar and quickly scare away hippopotamuses and elephants that eat our crops’. (Male, ward councillor, 54 years)

The raised structures vary in size and purpose. In order to serve whatever purpose, the structures are flood-proofed by elevating their floor system above the likely flood level, usually 3–5 m. The uninhabitable part of the structure is made resistant to flood damage and allows water to pass through. This practice is a commendable wet-proofing strategy, as it keeps the grain beyond the reach of floodwaters. The key informant further explained that other structures were anchored on the ground with mopane trees (*Colophospermun mopane* and *Julbernardia globiflora* species) or logs. They narrated stories of many villagers who survived floods by seeking shelter in such structures for two or three consecutive days during the 2015 floods. Similarly, Mavhura et al. (2013) found that villagers in flood-prone areas usually apply indigenous construction techniques that prevent water from reaching the plinth level of their dwellings during flood events. The techniques involve building houses on raised land or earthen platforms. Paul and Routray (2010) support the use of housing material that is easily transferrable and not susceptible to flooding.

Smallholder farmers also developed a dual home system: one in low-lying areas and another outside the floodplain. When faced with floods, the smallholder farmers temporarily migrate from low-lying areas to their second home. After floods, they return to their floodplain home and continue practising *mudzedze*. Although the dual home system is a flood coping measure, it remains a risky practice, especially when the smallholder farmers return to the unsafe floodplains. Even the flood-based farming system remains a fragile and unsustainable form of livelihood. As the PAR model shows, the farmers are at high risk to flood hazards. To reduce their vulnerability, 85% of the interviewees admitted that smallholder farmers grow drought-resistant crops such as millet, cotton and *rapoko*. These crops are grown outside the floodplain, where there is less exposure to floods.

At the same time, 90% of the key informants admitted having used plastic containers to store drinking water before, during and after floods. The need for clean drinking water stems from the observations that post-flood diseases spread through contaminated drinking water. The villagers are aware of the risk of cholera and typhoid that spread through shallow wells and submerged boreholes. Because of poverty, most of the survivors cannot afford to buy pure water.

The FGD participants also divulged that non-structural measures were commonly used to reduce vulnerability to floods. These measures include flood forecasting and early warning/action. The flood forecasting and early warning measures rely mainly on traditional knowledge through observing migratory birds such as *mashuramurove (ciconia ciconia)*. One village headman indicated the following:

‘The appearance of mashuramurove (*ciconia ciconia*) birds in November/December signifies above normal rains and the possibility of flooding. Also, the abundance of wild fruits like hwaka (*strychnos* madagascar-rensis) signifies potential flooding due to torrential rainfall. When we observe these things, community members move inland’. (Male, village head, 77 years)

Research has shown that both early warning and disaster preparedness can reduce disaster impact, especially if the two are accompanied by early action such as evacuation and relocation (Mabuku et al. 2018; Noorhashirin et al. 2016).

## Conclusion and policy implications

This study has explored perspectives of flood survivors on vulnerability to floods in Mbire district, Zimbabwe. It has also examined survivors’ views on vulnerability reduction measures. It has emerged that vulnerability to flooding is driven by a shortage of land, flood-based farming practices, and poverty and climate changes, amongst others. These factors increase smallholder farmers’ exposure and susceptibility to floods in different ways. Therefore, flood hazards in Mbire are both natural and induced by humans. The most affected groups of people include women, children and the elderly. To reduce their vulnerability, the smallholder farmers mainly rely on traditional flood-proofed structures, dual home system, early warning and early action. The communities in Mbire are aware of the flood risk they face and its causes. However, they have limited capacities in reducing their vulnerability conditions. These conditions are mainly caused by prevailing high poverty and overreliance on rain-fed farming in an area characterised by dry spells and drought.

Given the above, there is a need to diversify smallholder farmers’ livelihoods beyond the farming sector. This diversification should be coupled with investment in irrigation infrastructure to move away from their dependency on rain-fed and flood-based farming systems. Human settlements should be constructed far away from unsafe floodplains to reduce the farmers’ exposure to flood risks. At the same time, flood-based farming should be discouraged through enforcing environmental laws and community education on the long-term negative impacts of recession farming. The case of high poverty levels points to the need for enhancing smallholder farmers’ capacities through livelihood diversification, increasing access to disposable income and social protection measures such as flood insurances. Research has shown that people are better able to protect themselves and prepare for disasters when their incomes are more than just a subsistence wage (McEntire 2012).
